# The Diagnosis, Localisation, and Surgical Treatment of Tumours Pressing on the Spinal Cord

**Published:** 1892-04-09

**Authors:** 


### SURGERY.

### THE DIAGNOSIS, LOCALISATION, AND SURGICAL TREATMENT OF TUMOURS PRESSING ON THE SPINAL CORD.

Since 1887, when Horsley removed a tumour pressing on the spinal cord, the diagnosis of slow compression of the cord by new growths, has received quite a new stimulus, for the possibility of surgical treatment has been demonstrated, and a disease producing often intense agony, and always a slowly approaching death, can be fairly well localised in the spine, and with strict antiseptic precautions, removed, with only moderate risk to life, and sometimes with complete cure.

Horsley's patient was a man, æt. 42, who, in June, 1884, suddenly began to suffer from pain just below the left scapula. This continued, with remissions for considerable periods, until February, 1887. Sometimes it was very intense, and it was increased by movement. Loss of power came on in the left leg in February, 1887, and very soon after in the right. In April sensibility became impaired, and urine was retained in the bladder. Dr. Gower's description of the condition of the patient in June* is as follows: "There was absolute palsy of the legs, and cutaneous sensibility of all kinds was lost as high as the ensiform cartilage. At and just above this level, *i.e.*, in the region of the sixth and seventh inter-costal nerves, he complained of severe pain around the chest, much more severe on the left side than on the right, and increased to evident agony on any movement. The legs from time to time became rigid in extensor spasm, and a clonus could be obtained with great readiness in the muscles of the calf and front of the thigh. The bladder was distended." On a later examination, on June 9th, when Mr. Horsley operated, loss of sensibility as high as, and involving destruction of the fifth dorsal nerve, was noted. There was no excurvation of the spine, but there was tenderness on pressure on the left side of the sixth dorsal spine.

A small fibro-myxoma was found attached to the posterior

* See the 71st vol. of "Medico Chirurgical Society's Transactions."

root of the fourth dorsal nerve, and greatly compressing the lateral aspect of the cord. The growth and nerve root were removed together. First, the fourth, fifth, and sixth dorsal spines were removed, but nothing was discovered; only after removing two more laminæ above, was the growth discovered four inches above the level of complete anæsthesia; and two nerve roots higher than the distribution of the pain which was noted by Dr. Gowers, as in the distribution of the sixth and seventh nerves. But the question of localisation will be more fully referred to further on. All the symptoms gradually subsided after the operation, and in November he was walking about with the aid of sticks, and in January could walk three miles, and the pain had quite gone; in fact, he was cured. However, for many weeks after the operation the pain was very severe. The power returned first in the limb last paralysed.

Horsley's second case died from shock.* Rehn records the successful removal of a "lymphangioma" from the spinal canal, which was pressing on the cauda equina, and caused paresis of the lower limbs, bladder, and rectum, with "neuralgia." Abbe removed an extradural sarcoma, but, like Horsley's second case, with fatal result. Thus, out of four cases two have recovered.

Horsley has collected the records of fifty-eight cases of tumours pressing on the cord, and by a careful analysis of these he has shown us what are the symptoms usually produced by these growths.

They may grow between the dura mater and the bony wall of the spinal canal. The commonest growths in this situation are lipomata, which grow from the loose fat between the dura mater and the bone; sarcomata; hydatid cysts; and tubercular growths. More commonly they are intradural, and such tumours usually are fibromata or myxomata, sarcomata, and psammomata or tubercular growths. Even when intradural, Horsley says their connection with the spinal cord is of the slightest, so that they can be dissected from it without causing the slightest injury to a single nerve fibre.

The average duration was two and a-half years; the longest (cases of myxoma), seven years.

As the tumour grows the cord becomes softened from pressure. When the softening involves any of the great tracts ascending and descending degenerations follow. Sometimes the softening spreads up or down the cord irrespective of these tracts. This, the writer considers, to be due to thrombosis of the great median medullary vessels.

The great point in diagnosis is the way in which the symptoms develope, and this is more uniformly marked in the case of intradural growths than extradural. Generally, pain is the first symptom complained of—pain generally diagnosed as rheumatic or simple neuralgia—and, indeed, so long as pain remains the only symptom, as sometimes it does for a long period, we cannot make a correct diagnosis. But after a time this pain is followed by loss of power and then by sensory failure and retention of urine, and the formation of bedsores, due to the gradual onset of a complete transverse lesion with rigidity and spasm. It is, however, in the way these symptoms usually develop that we find the clue to diagnosis. First, pain (the distribution of which will be considered presently in connection with localisation), then motor weakness, then sensory failure. More important still when present, as it sometimes is, is the unilateral onset of the paralysis. Generally, as far as can be ascertained from the records which are not very complete on this point, the weakness begins from above downwards. The patient is unable to stand before the onset of paresis of the leg when lying down. The loss of sensibility spreads up the limbs from the soles of the feet. In most of the fifty-eight cases an examination of the spine gave no sign of disease. In only six was the diagnosis settled by the presence of an external tumour, solid or containing fluid. Tenderness on percussion, stiffness and weakness, and lateral curvature were sometimes noticed.

* B. Med. Jour. Dec. 6th, 1890.

And now with regard to the localisation of the lesion. The pain is invariably below the lesion, never above. But the lesion may be considerably above the pain. In the case Mr. Horsley operated on the pain was in the distribution of the sixth and seventh dorsal nerves, and the lesion was on the root of the fourth. It was also four inches above the level of anæsthesia. In the other cases the growth was situated at the level of the third and fourth dorsal vertebræ respectively, and the pain was in the lower limbs. In one case, however, the tumour grew on the posterior nerve root of the nerve in the distribution of which the pain was felt. Horsley says: "If the pain be extremely well defined in the course of one nerve of one side then a lesion may be diagnosed to exist in the course of that nerve, and so it may be localised with ease; and that where there is no delimitation of the pain, but where the anæsthesia is apparently well marked and in accord with nerve supply it is necessary to be most cautious and to define if possible the upper border of the hyper, or paræsthetic one which usually surrounds the upper limits of anæsthesia." He says we should always explore the highest point suggested by any definite symptom, but in his own case it was only on going higher that the tumour was found. A subjective feeling of weakness heightened by fatigue at one spot in the spine he regards as of considerable localising value.

A tumour in the *dorsal region* is always higher than tenderness over the spine. Lateral curvature is produced by tonic contraction of the muscles on the same side as the growth.

Horsley opens up the spinal canal by cutting off the spinous processes close to their base with bone forceps, and then trephining the laminæ. The canal is then further opened up by cutting the laminæ above and below. If nothing is found outside the dura mater, it is opened and the cord explored on all sides by passing an aneurism needle around. If necessary we can remove one dorsal nerve root, but in the lumbar enlargement the roots are so close together and so important we might be placed in much greater difficulties in our exploration of the front of the cord or the removal of a growth. It appears from experiments to be best to suture the dura mater, and not to leave a deep drainage tube in long, as a sinus discharging cerebro spinal fluid is apt to form along its track. That the operation should be done antiseptically is of course urgently needful.

